# Test–retest reliability of the 20-min pad test with infusion of strong-desired volume in the bladder for female urodynamic stress incontinence

**DOI:** 10.1038/s41598-020-75567-8

**Published:** 2020-10-28

**Authors:** Wen-Yih Wu, Sheng-Mou Hsiao, Pei-Chi Wu, Ho-Hsiung Lin

**Affiliations:** 1 Department of Obstetrics and Gynecology, Far Eastern Memorial Hospital, Banqiao, New Taipei Taiwan; 2 Graduate School of Biotechnology and Bioengineering, Yuan Ze University, Taoyuan, Taiwan; 3 Department of Obstetrics and Gynecology, National Taiwan University College of Medicine and Hospital, No. 8, Chung-Shan South Road, Taipei, 100 Taiwan

**Keywords:** Medical research, Urology

## Abstract

The repeatability of the 20-min pad test has not been reported. The aim of this study was to evaluate the test–retest reliability of the 20-min pad test in women with urodynamic stress incontinence. Among 89 enrolled women, 67 (75%) women were diagnosed with urodynamic stress incontinence and were examined in this study. The mean strong-desire volume of all the women was 306.7 ± 115.7 mL. The pad weights of the test versus retest were 28.3 ± 41.2 g versus 28.4 ± 38.6 g, p = 0.29, respectively. The test and retest pad weight results had a Spearman’s rho of 0.788 (*p* < 0.0001). The intraclass correlation coefficient was 0.793 (95% confidence interval, 0.704–0.882; *p* < 0.0001). The Bland–Altman plots all revealed good agreement between the test and the retest in the pad weights. In conclusion, the 20-min pad test infused with a strong-desired volume has good test–retest reliability to assess the severity of urine leakage for women with urodynamic stress incontinence.

## Introduction

The pad test was initially proposed by Sutherst et al. in 1981^1^ and modified by the Standardization Committee of the International Continence Society (ICS)^2,3^. It is a noninvasive method of quantifying the volume of urine leakage in patients with urinary incontinence^4^. There are two versions: the short-term pad test, which is performed in the clinic over a period of one hour, and the long-term pad test, which is usually performed at home over a period of 24 h^4,5^. The most commonly used protocol of the pad test is the standardized ICS one-hour pad test protocol, starting with 500 mL drinking water without voiding^3^. Thus, the doctors cannot know the exact urine volume in the bladder when the test begins.

The20-min pad test was proposed by Hahn and Fall^6^ and modified by Sand and Ostergard since 2005^7^. The 20-min pad test is performed with artificial water infusion into the bladder rather than by natural diuresis as in a 1-h pad test^7^. The 20-min pad test was reported to have a better sensitivity than the one-hour pad test for women with stress urinary incontinence (SUI)^8^. In addition, the strong-desired (SD) volume was reported to have a better sensitivity than 250 mL as the volume of water infusion before the initiation of the 20-min pad testing^9^.

A significant variation between the test and the retest and inadequate repeatability of the one-hour pad test has been reported^10,11^. As mentioned above, the 20-min pad test has been reported to have a better sensitivity than the 1-h pad test^8^. However, the repeatability of the 20-min pad test has not been reported. We were interested in whether the 20-min pad test had adequate repeatability. Thus, the aim of this study was to elucidate the test–retest reliability of the 20-min pad test.

## Results

After urodynamic studies, 67/89 (75%) of women with SUI were diagnosed as having urodynamic stress incontinence (USI) and received a retest of the 20-min pad testing. According to the presence of detrusor overactivity (DO), these 67 women with USI were divided into the USI only group (n = 53) and the USI and DO group (n = 14). The USI with DO group had a lower volume at first desire, normal desire and SD to void compared with the USI only group (Table 1).

**Table 1:** Baseline characteristics and urodynamic parameters of women with urodynamic stress incontinence and subgroups.

Variables	All women (n = 67)	USI (n = 53)	USI + DO (n = 14)	P^†^
Age (years)	53.9 ± 9.9	54.6 ± 10.5	51.4 ± 6.6	0.34
Parity	2.9 ± 1.4	2.9 ± 1.3	2.8 ± 2.0	0.23
Pad weight–test (g)	28.3 ± 41.2	26.1 ± 39.5	36.7 ± 47.9	0.28
Pad weight–retest (g)	28.4 ± 38.6	25.2 ± 35.5	40.6 ± 48.1	0.21
Maximum flow rate (mL)	26.2 ± 12.7	26.5 ± 13.2	25.1 ± 10.9	0.87
Average flow rate (mL)	9.1 ± 5.8	9.5 ± 6.1	7.6 ± 4.0	0.36
Voided volume (mL)	352.4 ± 186.5	370.0 ± 194.7	286.0 ± 137.4	0.22
Postvoid residual volume (mL)	104.2 ± 72.7	110.3 ± 77.1	81.1 ± 48.3	0.28
Voiding time (s)	113.2 ± 120.6	99.1 ± 88.1	166.6 ± 197.4	0.06
First desire (mL)	171.2 ± 62.7	180.3 ± 64.9	136.9 ± 39.2	0.01
Normal desire (mL)	228 ± 85.1	240.3 ± 89.1	181.6 ± 45.9	0.02
Strong desire (mL)	306.7 ± 115.7	324.7 ± 118.6	238.7 ± 73.5	0.01
Urgency to void (mL)	365.7 ± 136.2	381.6 ± 135.9	305.3 ± 123.9	0.055
Infused volume during pad test (mL)	305.9 ± 123.7	313.5 ± 130.6	277.2 ± 91.2	0.049
Infused volume during pad retest (mL)	291.9 ± 116.3	306.1 ± 121.6	237.9 ± 73.9	0.050

*DO* detrusor overactivity, *USI* urodynamic stress incontinence.

^†^Wilcoxon ranksum test was performed to compare the USI versus the USI + DO groups.

**Figure 1:**
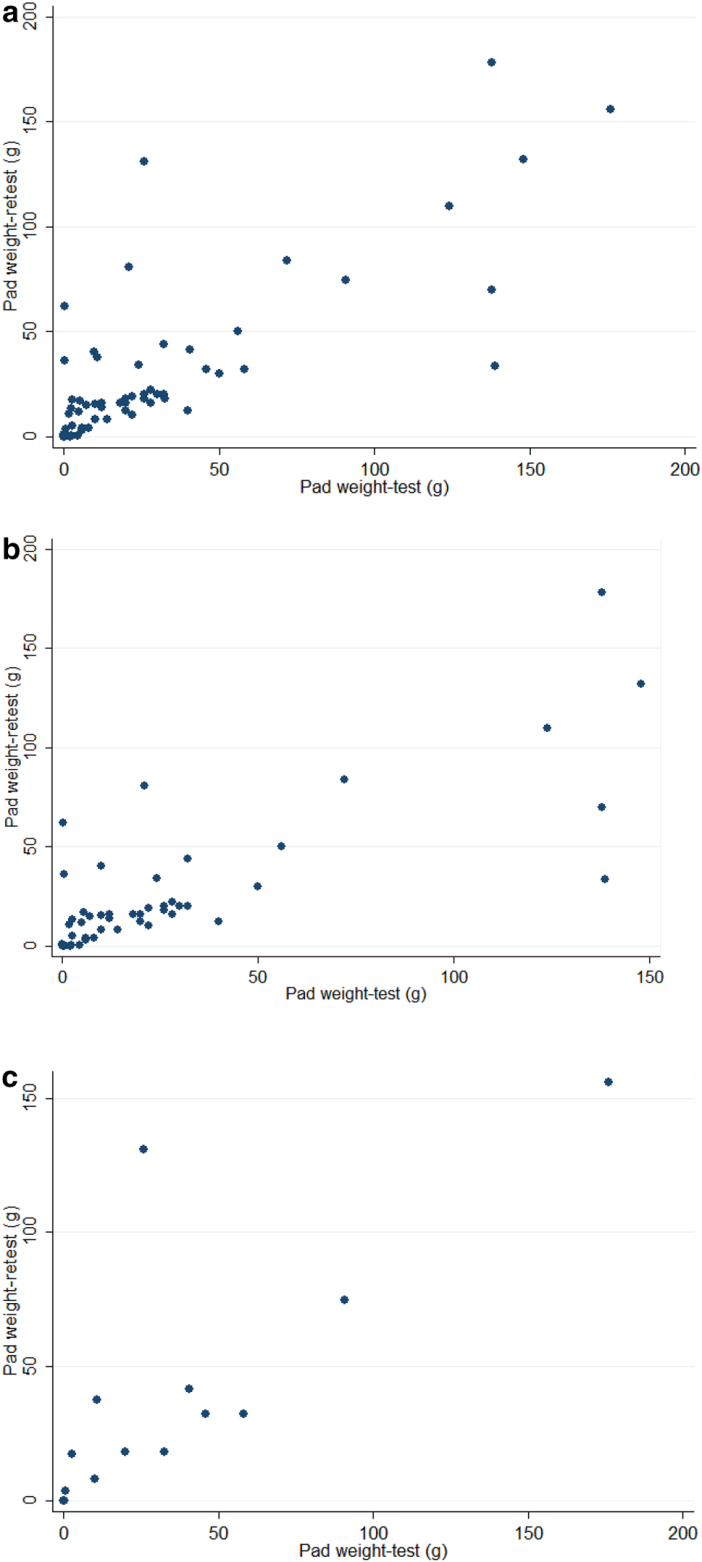
Scatter plots of the pad weight-test vs. the pad weight-retest in (**a**) all women, (**b**) women with urodynamic stress incontinence only and (**c**) women with both urodynamic stress incontinence and detrusor overactivity.

The pad weights of the test and the retest in all 67 women, the USI only group and the USI and DO group are shown in Table 2. The distribution of pad weights of the test and the retest in all women and both subgroups are shown in Fig. 1.

**Table 2:** Comparisons of test and retest results of 20-min pad test infused with the strong-desired volume of distilled water in the bladder of women with urodynamic stress incontinence.

Variables	n	Pad weight-test (g)	Pad weight-retest (g)	P^†^	Spearman correlation (rho)	P^‡^	Intraclass correlation coefficient	P^§^
All women	67	28.3 ± 41.2	28.4 ± 38.6	0.29	0.788	< 0.0001	0.793 (0.704–0.882)	< 0.0001
USI	53	26.1 ± 39.5	25.2 ± 35.5	0.40	0.761	< 0.0001	0.797 (0.698–0.896)	< 0.0001
USI + DO	14	36.7 ± 47.9	40.6 ± 48.1	0.66	0.848	0.0001	0.785 (0.581–0.989)	0.0002

*DO* detrusor overactivity, *USI* urodynamic stress incontinence.

^†^Wilcoxon signed-rank test.

^‡^Spearman correlation.

^§^Intraclass correlation coefficient.

**Figure 2:**
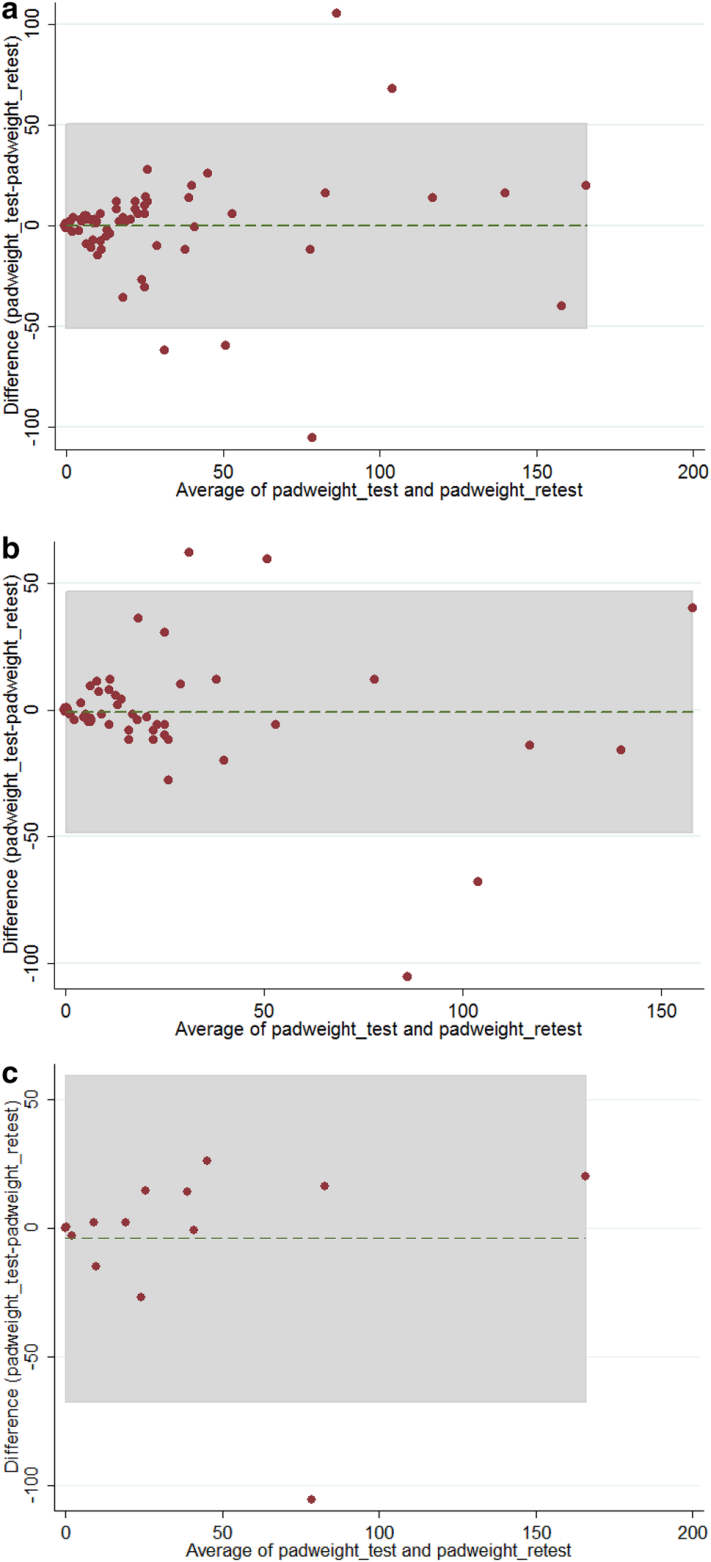
The Bland–Altman plots of the average and the differences of the pad weight–test and the pad weight-retest in (**a**) all women, (**b**) women with urodynamic stress incontinence only and (**c**) women with both urodynamic stress incontinence and detrusor overactivity.

The Wilcoxon signed rank test revealed no difference in pad weights between the test and the retest in all women (*p* = 0.29), the USI only group (*p* = 0.40), and the USI and DO group (*p* = 0.66). In addition, the Spearman correlation of pad weights between the test and the retest was 0.788 (*p* < 0.0001) in the 67 women (Fig. 1a). The Bland–Altman plot revealed a mean difference of − 0.125 (confidence interval [CI]: − 6.420 to 6.179) for the test vs. retest results (limits of agreements: − 50.786 to 50.536) (Fig. 2a). There was no difference in variances (Pitman’s test, r = 0.106, p = 0.393).

In addition, the subgroup analysis also revealed a good rest and retest correlation in the pad weight of the USI only group (rho = 0.761, *p* < 0.0001, Fig. 1b). The Bland–Altman plot revealed a mean difference of 0.881 (CI: − 5.772 to 7.534) for the test vs. retest results (limits of agreements: − 48.188 to 46.426) (Fig. 2b); and there was no difference in variances (Pitman’s test, r = 0.172, p = 0.219).

The subgroup analysis also showed a good rest and retest correlation in the pad weight of the USI and DO group (rho = 0.848, *p* = 0.0001) (Fig. 1c, Table 2). The Bland–Altman plot revealed a mean difference of − 3.936 (CI: − 22.572 to 14.701) for the test vs. retest results (limits of agreements: − 67.199 to 59.328) (Fig. 2c); and there was no difference in variances (Pitman’s test, r = − 0.007, p = 0.981). The intraclass correlation coefficients of the pad weights between the test and the retest were 0.793 (95% CI 0.704–0.882; *p* < 0.0001) in all 67 women with USI, 0.797 (95% CI 0.698–0.896; *p* < 0.0001) in the USI only group, and 0.785 (95% CI 0.581–0.990; *p* = 0.0002) in the USI and DO group (Table 2).

Pad weight > 1 g was defined as the positive result, and there was no significant difference in the results between the test and retest pad tests (n = 67, McNemar’s test, p = 0.48). The agreement rate was 88.1%. The kappa statistic showed a substantial agreement (kappa value = 0.62, 95% CI 0.38–0.86).

Similarly, in the USI only group, there was no significant difference in the results between the test and retest pad tests (n = 53, McNemar’s test, p = 0.26). The agreement rate was 86.8%. The kappa statistic showed a moderate agreement (kappa value = 0.59, 95% CI 0.31–0.86).

In the USI and DO group, there was no significant difference in the results between the test and retest pad tests (n = 14, McNemar’s test, p = 0.32). The agreement rate was 92.9%. The kappa statistic showed a substantial agreement (kappa value = 0.76, 95% CI 0.32–1.00).

## Discussion

Significant variation between the test and retest and inadequate repeatability of the one-hour pad test have been reported^10,11^. Nonetheless, in our study, pad weights in the test and retest of the 20-min pad testing revealed a significant Spearman’s correlation (Table 2), good agreement in the Bland–Altman plot (Fig. 2), a good intraclass correlation (Table 2), and no difference in the Wilcoxon signed rank test (Table 2). Thus, our study revealed that the 20-min pad test should have a good test–retest reliability for the quantification of urine leakage for female patients with USI.

In Table 1, women in the USI and DO group tended to have a smaller bladder capacity with a mean infused volume of less than 250 mL (mean: 238.7 mL), while the USI only group had a mean infused volume of more than 250 mL (mean 324.7 mL, Table 1). It has been reported that bladder infusion with an SD volume of water has better sensitivity than bladder infusion with 250 mL before the beginning of the 20-min pad testing^9^. Our study confirmed that if we infused 250 mL into the bladder for pad testing in each USI woman, some women would have bladder overdistension or inadequate distension, resulting in excessive or decreased urine leakage during pad testing, leading to overestimation or underestimation of the SUI severity. The above findings may support the findings of better sensitivity of bladder infusion with the SD volume of water for a 20-min pad test compared with 250 mL^9^.

Pad testing is used to assess SUI severity^4^. In this study, the determination of the SD volume was derived from filling cystometry, which is an invasive examination. However, we can estimate the SD volume from the bladder diary without the need for filling cystometry. It had been reported that excluding the first morning void, the days 1 to 3 average maximum daytime voided volumes in the bladder diary had good correlation with the SD volume^12^. In addition, on day 1, the maximum daytime voided volumes have been shown to be well correlated with the SD volume^12^. Thus, we can request that our patient record the bladder diary for at least one day before the 20-min pad testing, and then we can derive the maximum daytime voided volume as a surrogate for the SD volume. Therefore, we can use the maximum daytime voided volume as the volume for bladder infusion before the initiation of pad testing. In addition, simply infusing distilled water until one feels that one must go to the restroom might be another alternative. Furthermore, a soft, thin, lubricated catheter is suggested to minimize the irritation to the urothelium of the urethra, which may induce more urine leakage or urethral contractility^13^.

Common female lower urinary tract disorders include SUI, overactive bladder syndrome (OAB), bladder outlet obstruction, bladder dysfunction and interstitial cystitis/painful bladder syndrome (IC/PBS)^14^. SUI is the complaint of involuntary loss of urine on effort or physical exertion, or on sneezing or coughing^15^, and is generally treated by surgery^16^. OAB is defined by the presence of urinary urgency, usually accompanied by frequency and nocturia, with or without urgency urinary incontinence, and in the absence of urinary tract infection or other obvious pathology^15^. OAB is frequently treated by pharmacology, such as antimuscarinics and beta-3 agonists^17^. Refractory OAB can be treated by sacral neuromodulation, transvaginal electrical stimulation or intravesical botox injection^18,19^. Based on the finding of good test–retest reliability of the 20-min pad test for women with USI with or without DO, the 20-min pad test seems to be a good tool to assess the severity of SUI with or without OAB.

IC/PBS is a complex pathology, which is often associated with vulvodynia, endometriosis and pelvic floor dysfunction in women^20^, and may present with urinary urgency, urgency incontinence, recurrent urinary tract infection, vaginal dryness or dyspareunia^21^. Thus, IC/PBS should be considered in the differential diagnosis for women with overactive bladder symptoms with pelvic organ discomfort/pain^21^. Pad tests have been used to assess the therapeutic effect in women with OAB^22,23^. Steps for performing a 20-min pad test include hand-washing^7^. Hand-washing may induce involuntary detrusor contraction, resulting in urgency incontinence^24^. Thus, the 20-min pad test might be used to assess urgency incontinence in women with IC/PBS.

Limitations of this study include the small sample size and the fact that the work was undertaken more than a decade ago. A larger prospective study may be proposed in the future.

In conclusion, the 20-min pad test infused with the SD volume of water has good test–retest reliability to assess the severity of urine leakage for women with USI.

## Methods

Between August 2007 and December 2010, all consecutive women with SUI who visited the urogynaecology outpatient clinics of a tertiary referral centre were invited to participate in this study. The exclusion criteria included urinary tract infection and chronic pelvic inflammation and without USI. Only women with USI were included in this study. This study was approved by the Research Ethics Committee of National Taiwan University Hospital. Informed consent was obtained from all participants. All methods in this study were performed in accordance with the relevant guidelines and regulations.

Each enrolled woman underwent a urodynamic study, including uroflowmetry, filling (with a rate of 60 mL H_2_O/min of 35 °C distilled water) and voiding cystometry, and stress urethral pressure profile. The SD volume for each patient was derived from the filling cystometry.

The 20-min pad test was first proposed by Hahn and Fall^6^ and modified by Sand and Ostergard^7^. The detailed steps are as follows. Each patient's bladder was emptied with a transurethral catheter and refilled with the SD volume of distilled water. The catheter was then removed, and the patient returned to a standing position with a preweighed perineal pad placed on the underwear. Thereafter, the patient was asked to cough 10 times, bear down 10 times, do 10 deep knee bends, jump up and down on the spot 10 times, wash her hands under cold water for 1 min, walk up and down five stairs 10 times, walk in the hall for 10 min, and then remove the perineal pad. The perineal pad was weighed, and the weight gain was derived by subtracting the original dry pad weight from the current pad weight. The positive pad weight result was defined as more than 1 g of weight gain^7,25^.

Because the main purpose of this study was to evaluate the test–retest reliability of the 20-min pad test for patients with USI, only patients with USI were requested to receive a retest of the 20-min pad test within one week after the previous pad test. The physical activities were the same in the test and retest of the 20-min pad testing.

In this study, USI was diagnosed if involuntary urine leakage was noted during filling cystometry, associated with increased intra-abdominal pressure and an absence of detrusor contraction. DO was diagnosed if involuntary detrusor contraction occurred during filling cystometry.

Multichannel urodynamic equipment (Life-Tech, Houston, TX, USA) with computer analysis and Urovision (Urolab Janus System V, Houston, TX, USA) was used. All terminology conformed to the standards recommended by the ICS^3^. All procedures were performed by an experienced technician, and the data were interpreted by a single observer to avoid interobserver variability.

STATA software (Version 11.0; Stata Corp, College Station, TX, USA) was used for the statistical analyses. The Spearman rank-correlation coefficient and intraclass correlation coefficient were tested for correlation and reliability of the test and retest results. The Wilcoxon signed rank test was tested for the difference of the test and retest. A *p* value of less than 0.05 was considered statistically significant. A Bland–Altman plot was plotted as the mean difference between the test and retest results against the average of the test and retest results to assess agreement between the two tests^26^. The limits of agreement were defined as the mean difference ± 1.96 X standard deviation. Pitman’s test was used to test the difference in variances^27^. Cohen’s kappa statistic is used to measure interrater reliability for categorical items^28^. Kappa statistic < 0.00 indicates poor agreement, 0–0.20 indicates slight agreement, 0.21–0.40 indicates fair agreement, 0.41–0.60 indicates moderate agreement, 0.61–0.80 indicates substantial agreement, and 0.81–1 indicates almost perfect agreement^29^.
